# A genome-wide association study demonstrates significant genetic variation for fracture risk in Thoroughbred racehorses

**DOI:** 10.1186/1471-2164-15-147

**Published:** 2014-02-21

**Authors:** Sarah C Blott, June E Swinburne, Charlene Sibbons, Laura Y Fox-Clipsham, Maud Helwegen, Lynn Hillyer, Tim DH Parkin, J Richard Newton, Mark Vaudin

**Affiliations:** 1 Centre for Preventive Medicine, Animal Health Trust, Lanwades Park, Kentford, Newmarket, Suffolk CB8 7UU, UK; 2 British Horseracing Authority, 75 High Holborn, London WC1V 6LS, UK; 3 Present address: Boyd Orr Centre for Population and Ecosystem Health, School of Veterinary Medicine, College of Medical, Veterinary and Life Sciences, University of Glasgow, 464 Bearsden Road, Glasgow G61 1QH, UK

**Keywords:** Fracture risk, Horse, Genome-wide association, Genetic variation

## Abstract

**Background:**

Thoroughbred racehorses are subject to non-traumatic distal limb bone fractures that occur during racing and exercise. Susceptibility to fracture may be due to underlying disturbances in bone metabolism which have a genetic cause. Fracture risk has been shown to be heritable in several species but this study is the first genetic analysis of fracture risk in the horse.

**Results:**

Fracture cases (n = 269) were horses that sustained catastrophic distal limb fractures while racing on UK racecourses, necessitating euthanasia. Control horses (n = 253) were over 4 years of age, were racing during the same time period as the cases, and had no history of fracture at the time the study was carried out. The horses sampled were bred for both flat and National Hunt (NH) jump racing. 43,417 SNPs were employed to perform a genome-wide association analysis and to estimate the proportion of genetic variance attributable to the SNPs on each chromosome using restricted maximum likelihood (REML). Significant genetic variation associated with fracture risk was found on chromosomes 9, 18, 22 and 31. Three SNPs on chromosome 18 (62.05 Mb – 62.15 Mb) and one SNP on chromosome 1 (14.17 Mb) reached genome-wide significance (*p* < 0.05) in a genome-wide association study (GWAS). Two of the SNPs on ECA 18 were located in a haplotype block containing the gene zinc finger protein 804A (*ZNF804A*). One haplotype within this block has a protective effect (controls at 1.95 times less risk of fracture than cases, *p* = 1 × 10^-4^), while a second haplotype increases fracture risk (cases at 3.39 times higher risk of fracture than controls, *p* = 0.042).

**Conclusions:**

Fracture risk in the Thoroughbred horse is a complex condition with an underlying genetic basis. Multiple genomic regions contribute to susceptibility to fracture risk. This suggests there is the potential to develop SNP-based estimators for genetic risk of fracture in the Thoroughbred racehorse, using methods pioneered in livestock genetics such as genomic selection. This information would be useful to racehorse breeders and owners, enabling them to reduce the risk of injury in their horses.

## Background

Metabolic bone disorders are often a cause of bone fragility and increased risk of fracture. A common example of a bone metabolic disorder in humans is osteoporosis; a late-onset disease characterized by low bone mineral density, structural deterioration of bone tissue and an elevated risk of fracture in affected individuals. Bone fragility has an estimated heritability of 16-54% [1-3] in humans, depending on fracture site and type, and several genes associated with bone mineral density and fracture risk have been identified in both humans and other species [4-7], although the genes underlying each of these traits appear to be different [8,9].

Bone fractures with non-traumatic origin occur in Thoroughbred racehorses, with the majority of fractures occurring in the distal limbs; bones subject to high impact and load during exercise and racing. Fracture is the main reason for euthanasia of horses on the racecourse [10], with an average of 60 horses per year suffering a fatal distal limb fracture during racing in the UK (both flat and National Hunt jump racing) [11]. The prevalence of all fatal and non-fatal fractures occurring during training is between 10-20% [12,13]. Studies of the pathology of equine fracture indicate evidence of stress-related damage to the bone prior to fracture, which may be related to metabolic disturbances in bone re-modelling [14,15].

Fracture risk has been demonstrated to be heritable in several species but its heritability in the horse has not been previously investigated. In this study, we have identified candidate genome regions associated with fracture risk in the Thoroughbred horse by carrying out a genome-wide association study (GWAS) with 43,417 SNPs genotyped on 269 fracture cases and 253 controls. We have also demonstrated that there is significant genetic variation for fracture risk in the Thoroughbred horse, distributed among several chromosomes.

## Methods

### Data

Fracture cases were horses that sustained catastrophic distal limb fractures while racing on UK racecourses, which necessitated euthanasia. A total of 276 fracture case samples were obtained from an archive of bone and tissue collected during a previous study between February 1999 and May 2005 [11]. The exact fracture site and type were identified by post-mortem examination. The frequency of fracture locations is shown in Additional file 1: Table S1. All fracture sites were in bones below and including the knee and hock. No cases with fractures in other bones, for example, the pelvis, neck or skull, were included in the study. The phenotype can, therefore, be regarded as a sub-set of fracture types involving only the distal limbs. Control samples (n = 269) came from a mixture of uninjured horses originally selected from the same race as the case (n = 66) and uninjured horses sampled as part of a previous study (n = 203). Control horses were over 4 years of age, were racing during the same time period as the cases, and had no history of fracture at the time the study was carried out. After genotyping quality control 522 horses remained in the analysis. Horses were bred for both flat and National Hunt (NH) jump racing: of the cases 135 were flat-bred, 110 NH-bred and 24 of unknown status, of the controls 117 were flat-bred, 135 NH-bred and 1 of unknown status (Table 1). Horse identities were anonymised, and no pedigree information was available.

**Table 1:** Distribution of fracture cases and controls by background and sex

	**Cases**	**Controls**
**Flat-bred**		
Male	104	103
Female	31	14
**National Hunt (NH)-bred**		
Male	83	116
Female	27	19
**Unknown status**		
Male	20	1
Female	4	0
**Total**	269	253

The numbers of cases and controls shown are for samples which passed genotyping quality control.

### DNA extraction and quantification

Samples consisted of either tissue or bone marrow biopsies (cases) or blood samples collected in EDTA (controls). DNA was extracted using Nucleon BACC DNA extraction kits by Gen-Probe Life Sciences Ltd. DNA samples were quantified in duplicate using Quant_iT PicoGreen dsDNA kits (Invitrogen, Carlsbad, CA) and 10% of the samples were run on a 1% agarose gel to check for the presence of high molecular weight DNA. DNA aliquots were adjusted to a concentration of 70 ng/ul for genotyping.

### SNP genotyping and quality control

Samples were genotyped with the Equine SNP50 BeadChip (Illumina, San Diego, CA) by Cambridge Genomic Services (University of Cambridge, UK). The Equine SNP50 BeadChip contains 54,602 SNP assays (average density one SNP per 43.2 kb) selected from the database of over one million SNPs generated during the sequencing of the horse genome (http://www.broadinstitute.org/mammals/horse). The initial association results indicated a genome-wide significant region on ECA 18. In order to try to refine the position (fine map) the location of the associated gene, an additional 78 SNPs located on ECA 18 between 61.89 Mb and 71.17 Mb (listed in Additional file 2: Table S2), and not present on the BeadChip, were genotyped using a GoldenGate assay (Illumina, San Diego, CA) by Gen-Probe Life Sciences Ltd.

The genotyping data were analysed with GenomeStudio software (Illumina, San Diego, CA). A cluster file was generated directly from the fracture dataset (n = 545) together with an additional 797 Thoroughbred samples genotyped at the same time. All genotyping data were clustered *de novo* for the 1,342 samples. The average SNP call frequency was 98.82%, with 150 SNPs not called. Nineteen samples (1.4%) had a call rate less than 95% and these were discarded. The remaining samples were then re-clustered. The average SNP call frequency had increased to 99.17%, with only 143 SNPs (0.26%) not called from the 54,602 on the EquineSNP50 BeadChip.

All SNPs were then subjected to a number of editing steps with GenomeStudio software, during which thresholds were applied for a number of metrics following the chip manufacturer’s guidelines. This resulted in the removal of 190 SNPs with low intensity data (AB R Mean), 1,265 SNPs with inadequately defined clusters (cluster separation), 2,279 SNPs with call rates less than 98%, 297 SNPs where the heterozygote cluster was not well separated from the homozygote clusters (AB T Mean), 119 SNPs where genotypes differ significantly from Hardy-Weinberg equilibrium and 51 SNPs where X chromosome SNPs were heterozygous in males. A total of 4,201 SNPs were removed. The mean call frequency in the remaining SNPs was 99.83%. Markers with a minor allele frequency (MAF) less than 2% were excluded from the analysis (n = 11,124), as were markers that failed the Hardy-Weinberg equilibrium test (*p* < 0.001) (n = 96), and markers with more than 10% of genotypes missing (n = 4,223): 43,417 SNPs remained in the analysis.

Further quality control procedures on the samples, such as estimation of sample gender based on X chromosome genotypes and identification of duplicated samples based on genotype identity, resulted in 39 samples out of 1,342 being discarded. From the fracture study samples 269 cases and 253 controls passed the quality control procedures and 7 cases and 16 control samples failed.

### Statistical analyses

#### Population stratification

Possible population stratification was assessed by calculating identity-by-state (IBS) sharing among all pairs of individuals. A permutation test for between group IBS differences (where the null hypothesis is no similarity between groups) showed a significant (*p* < 2 × 10^-5^) degree of similarity (IBS) between cases and controls. There was also significant IBS between flat-bred and National Hunt-bred horses (*p* < 1 × 10^-5^), with evidence for more similarity within the flat-bred and National Hunt-bred groups than between the groups (within-group *p* < 1 × 10^-5^, between-group *p* < 7 × 10^-5^). These results suggest that both the cases and controls are drawn from genetically related populations. Similarly flat-bred and National Hunt-bred horses are from related populations, although there is some clustering (probably of family groups) within the flat and National Hunt populations. Additional file 3: Figure S1 shows multi-dimensional scaling plots based on IBS sharing between cases and controls, and flat-bred and National Hunt-bred horses. Cases and controls are evenly distributed through both the flat and National Hunt-bred populations.

#### Whole genome Cochran-Mantel-Haenszel (CMH) association

The Cochran-Mantel-Haenszel (CMH) association test (2 × 2 × K, where K = 52 clusters) in PLINK v1.07 [16] was used in order to correct for the potential confounding of population stratification (ppc 0.001). The CMH association test allows for comparison of cases and controls while controlling for clusters within the data, where the clusters are defined by IBS sharing among individuals (52 clusters were identified in the fracture data set). The CMH analysis tests each single SNP independently. Empirical *p*-values were calculated using 1,000 permutations with the adaptive permutation option in PLINK v1.07 [16].

#### Haplotype logistic regression analysis of ECA 18

Haplotype blocks in the region of interest on ECA 18 were identified based on the value of r^2^ and visualized using HAPLOVIEW [17]. Blocks containing significant SNPs were further analysed using haplotype logistic regression with PLINK v1.07, with sex and background (flat/National Hunt-bred) fitted as co-variates. Corrected *p*-values for the logistic regressions were obtained with 10,000 permutations. Haplotype frequencies within case and control groups were also determined.

#### Estimation of the genetic variance due to SNPs

Estimates of the genetic variance explained by all SNPs and by SNPs on individual chromosomes were obtained with Restricted Maximum Likelihood (REML) analysis using the GCTA program [18]. GCTA allows the proportion of variance explained by SNPs to be estimated for a complex disease based on case-control genome-wide association study (GWAS) data [19]. The method takes account of the binary (0-1) nature of case-control data and estimates the genetic variance on the, more interpretable, underlying liability scale. It also takes account of the bias in ascertainment, due to the proportion of cases being larger than the disease prevalence in the population.

A genetic relationship matrix was derived with GCTA [18,19] from the 43,417 genotyped SNPs. Sex was fitted as a fixed effect, as it had previously been determined to be a significant effect with an ANOVA analysis. GCTA accounts for relationships among individuals through the genetic relationship matrix, but also permits principal component analysis (PCA) eigenvectors to be included as covariates to capture variance due to population structure. In this analysis the first 20 eigenvectors were included as co-variates, in order to account for the structure of the flat and National Hunt-bred populations.

## Results and discussion

### Population stratification

IBS sharing among individuals indicated there was some genetic differentiation between the flat and National Hunt-bred horses, with clustering of families within the groups (Additional file 3: Figure S1 a and b). Pedigree analysis of 120 leading (ranked by offspring earnings) flat and NH sires in the UK in 2012 showed the mean coancestry among the sires was 0.0295, minimum 0.0024 and maximum 0.276. This confirms the existence of gene flow or shared ancestry between the groups. A multidimensional scaling plot derived from the pedigree-based coancestry (Additional file 3: Figure S1 c) shows a similar pattern to the DNA-based IBS sharing; there is family clustering within NH or flat-bred groups, but also evidence for some differentiation between the family lines used to produce flat or NH horses. In addition to genetic differences between the populations there are also differences in the environmental risks experienced. The risk of fracture depends on the type of racing: flat turf racing is the safest (0.4 fatal fractures/1000 starts) whilst National Hunt racing is associated with the highest risk (2.2 fatal fractures/1000 starts) [11]. The increase in environmental risk for National Hunt racehorses could make the ascertainment of genetically susceptible horses from this population more difficult, potentially decreasing the power of a genetic study.

Prior to correction for population stratification the genomic inflation factor (λ) for the genome-wide association study was 1.15, and after correction with the CMH test λ was 1.05. Additional file 4: Figure S2 shows the quantile-quantile (Q-Q) plot obtained after the CMH analysis. The reduction in λ suggests the CMH analysis is effectively accounting for population structure within the data. Inclusion of background (National Hunt or flat-bred) into a genome-wide logistic regression model gave a genomic inflation factor (λ ) of 1.04, also suggesting this classification of horses and its inclusion as a co-variate in the analysis corrects sufficiently for population stratification. Classification of background (with two levels) as a fixed effect in the logistic model is simpler than including PCA eigenvectors or CMH clusters where there are many levels (in this case, either 20 or 52), and thus permits large numbers of permutations to be computed in a reasonable time-frame.

### Genome-wide association study

**Figure 1:**
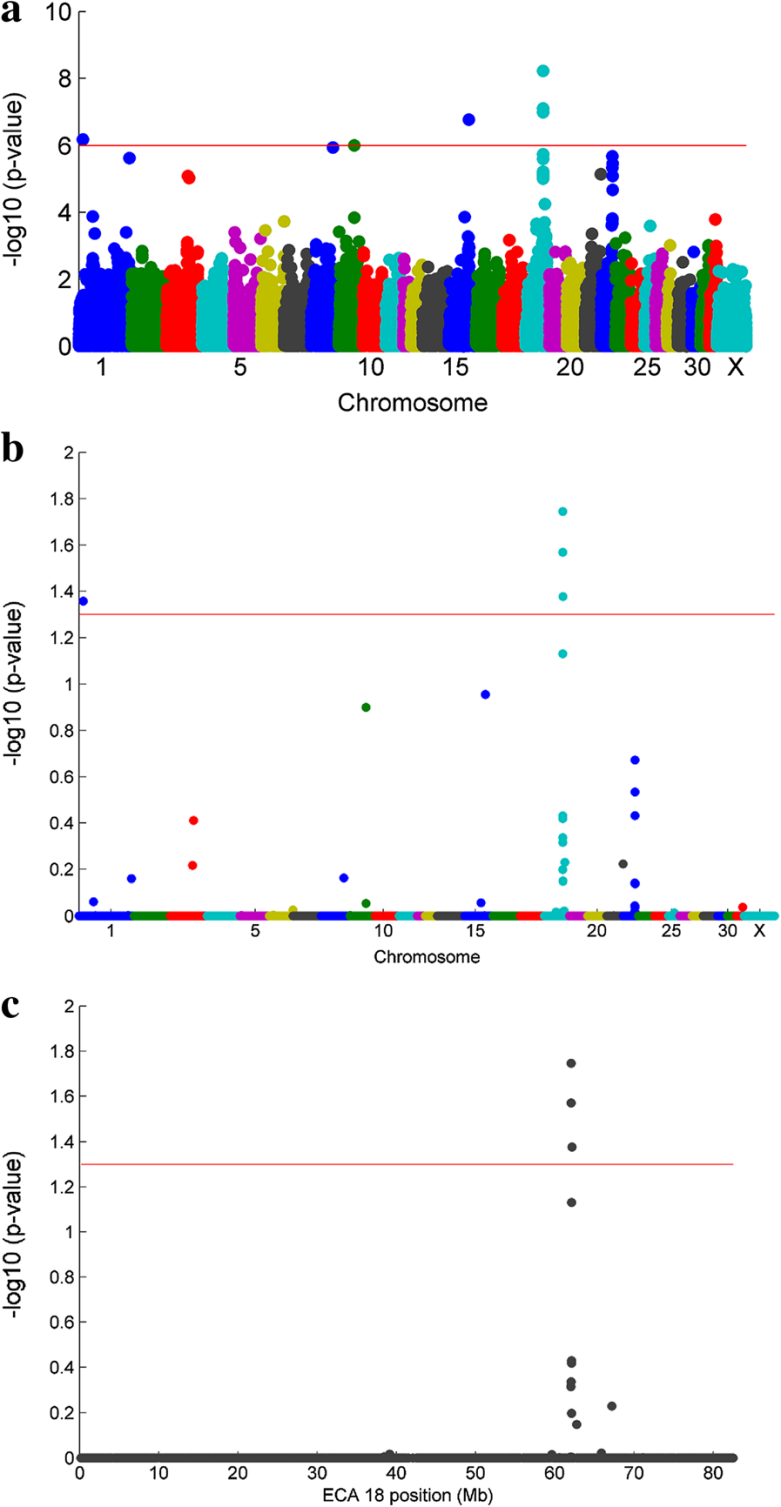
**Manhattan plots of raw and corrected *p*-values from the genome-wide association (CMH test) for whole genome and ECA 18. (a)** Manhattan plot of raw *p*-values from the genome-wide association analysis (CMH test) with flat and National Hunt-bred horses combined. **(b)** Manhattan plot of empirical *p*-values, calculated after 1000 permutations **(c)** empirical *p*-values for ECA 18 plotted against SNP position on the chromosome (Mb). The 5% genome-wide significance threshold is shown as a red line.

Three SNPs on ECA 18 and one on ECA 1, reached genome-wide significance after correction for multiple testing (*p*_genome_ < 0.05) Table 2. ECA 18 showed evidence for more than one SNP associated with distal limb fracture. A number of supporting SNPs are seen, with the peak localizing to around 62 Mb. There is also evidence of suggestive signals seen on ECA 3, 8, 9, 15, 21 and 22 although they do not reach genome-wide significance level. Figure 1 shows Manhattan plots of (a) the raw *p*-values from the genome-wide association (Cochran-Mantel-Haenszel) scan for distal limb fracture (b) empirical *p*-values, calculated after 1000 permutations (c) empirical *p*-values for ECA 18. The additional 78 SNPs on ECA 18, genotyped for fine mapping purposes, showed no significant associations with fracture risk and did not explain any more of the genetic variation in the heritability analysis than the SNPs included on the Equine SNP50 BeadChip.

**Table 2:** **Unadjusted (raw) and corrected *p*-values for the 25 top-ranking SNPs from the genome-wide Cochran-Mantel-Haenszel (CMH) association analysis**

**CHR**	**SNP**	**BP POSN**	**RISK ALLELE**	**FREQ CASES**	**FREQ CONTROLS**	**χ**^**2**^	**P**_**unadjusted**_	**OR**	**SE**	**P**_**corrected**_
**1**	**BIEC2-6883**	**14167279**	**G**	**0.389**	**0.274**	**21.44**	**3.66 × 10**^**-6**^	**1.99**	**0.153**	**0.044**
1	BIEC2-21406	50074740	G	0.878	0.821	14.60	1.33 × 10^-4^	2.13	0.197	0.868
1	BIEC2-85595	176967404	T	0.781	0.696	15.75	7.24 × 10^-5^	1.82	0.156	0.694
3	BIEC2-792745	73658246	T	0.955	0.911	16.16	5.82 × 10^-5^	3.20	0.298	0.608
3	BIEC2-793836	76584425	G	0.671	0.522	17.45	2.95 × 10^-5^	1.82	0.145	0.389
8	BIEC2-1062740	73825113	G	0.732	0.650	15.78	7.11 × 10^-5^	1.84	0.154	0.689
9	BIEC2-1094991	53231490	G	0.934	0.874	19.94	7.99 × 10^-6^	2.88	0.250	0.126
15	BIEC2-316813	68450494	A	0.937	0.864	20.15	7.17 × 10^-6^	2.99	0.256	0.111
18	BIEC2-438205	62035484	C	0.257	0.157	16.88	3.98 × 10^-5^	2.13	0.186	0.484
**18**	**BIEC2-416680**	**62049912**	**G**	**0.263**	**0.144**	**22.79**	**1.81 × 10**^**-6**^	**2.33**	**0.179**	**0.027**
**18**	**BIEC2-416681**	**62054146**	**A**	**0.265**	**0.144**	**23.74**	**1.10 × 10**^**-6**^	**2.38**	**0.180**	**0.018**
18	BIEC2-438210	62054710	C	0.255	0.157	17.02	3.70 × 10^-5^	2.13	0.186	0.462
18	BIEC2-416683	62115222	A	0.257	0.142	20.81	5.08 × 10^-6^	2.26	0.181	0.074
18	BIEC2-438214	62115702	T	0.259	0.153	17.49	2.88 × 10^-5^	2.17	0.187	0.382
18	BIEC2-438222	62118622	T	0.249	0.151	16.02	6.25 × 10^-5^	2.11	0.188	0.634
18	BIEC2-438227	62120598	C	0.255	0.153	17.53	2.83 × 10^-5^	2.17	0.188	0.372
**18**	**BIEC2-416704**	**62148769**	**G**	**0.255**	**0.139**	**21.81**	**3.00 × 10**^**-6**^	**2.32**	**0.182**	**0.042**
18	BIEC2-416766	62757935	A	0.466	0.322	15.66	7.60 × 10^-5^	1.77	0.144	0.710
18	BIEC2-417495	67186093	G	0.314	0.184	16.24	5.59 × 10^-5^	1.91	0.163	0.589
21	BIEC2-574084	57039393	G	0.865	0.778	16.19	5.72 × 10^-5^	2.10	0.187	0.599
22	BIEC2-595969	38591767	C	0.790	0.674	15.51	8.21 × 10^-5^	1.90	0.162	0.724
22	BIEC2-596079	38680459	G	0.799	0.673	18.78	1.47 × 10^-5^	2.04	0.165	0.213
22	BIEC2-596530	39157841	C	0.706	0.575	17.53	2.82 × 10^-5^	1.84	0.147	0.371
22	BIEC2-596542	39169750	C	0.710	0.575	18.10	2.10 × 10^-5^	1.86	0.147	0.293
22	BIEC2-596546	39170079	A	0.671	0.547	15.48	8.35 × 10^-5^	1.76	0.143	0.728

Genome-wide significant SNPs are shown in bold text. Corrected *p*-values were derived using 1000 permutations.

### Linkage disequilibrium and haplotype-block analysis

**Figure 2:**
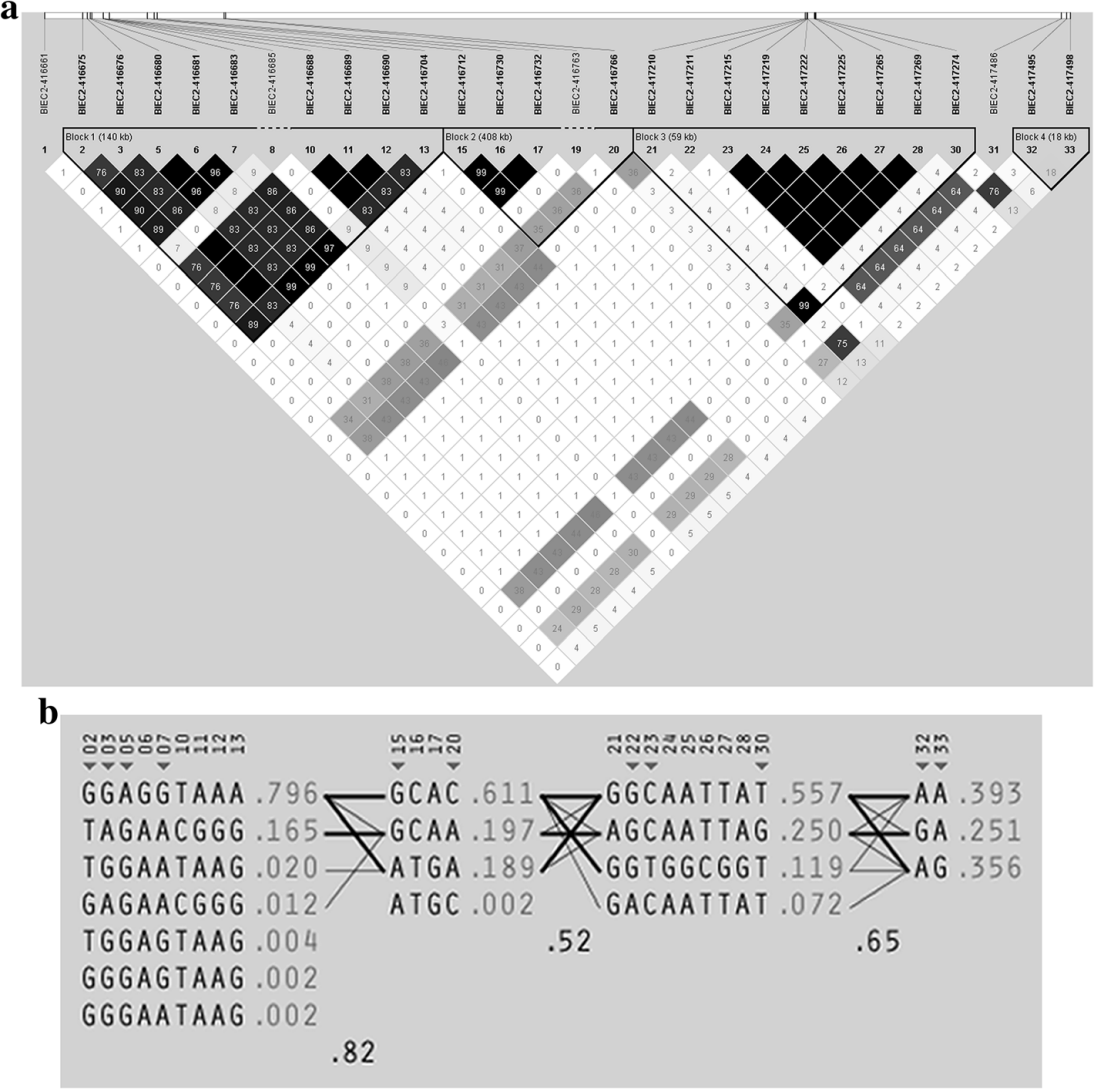
**Linkage disequilibrium (LD) and haplotype frequencies for LD blocks around significant SNPs on ECA 18. (a)** Linkage disequilibrium (LD) around significant SNPs on ECA 18. Haplotype block 1 (62.01 – 62.15 Mb) contains the two most significant SNPs from the genome-wide association study (BIEC2-416680 and BIEC2-416681). There is only one known gene within this haplotype block, *ZNF804A*. Haplotype block 2 (62.15 – 62.76 Mb) contains the candidate gene *FSIP2*, while haplotype block 3 (62.76 – 65.87 Mb) contains the candidate genes *ITGAV*, *CALCRL*, *COL3A1* and *COL5A2*. SNP BIEC2-417495 in haplotype block 4 (67.18 – 67.20 Mb) is in linkage disequilibrium (r^2^ = 0.8) with the myostatin (*MSTN*) gene, believed to be associated with racing performance [24-26], but there is only moderate LD (r^2^ < 0.3) between this SNP and the SNPs in haplotype block 1 which are significantly associated with catastrophic fracture risk **(b)** Observed haplotypes and their frequencies for the four haplotype blocks observed in the ECA 18 fracture associated region.

Examination of the linkage disequilibrium (LD) among SNPs showed that the most significant SNPs on ECA 18 fall into an LD block containing 10 SNPs in total and spanning 140 kb (haplotype block 1 in Figure 2). All SNPs within the block are in high LD with each other with pair-wise r^2^ of at least 0.76. The haplotype GGAGGCTAAA is at higher frequency in the controls and has a protective effect, with logistic regression (Table 3) showing that controls are 1.95 times at less risk of fracture than cases (*p* = 1 × 10^-4^). TGGAATTAAG, a risk haplotype, is at low frequency in the cases and, at least in this data set, absent from the controls. Cases with this haplotype are at 3.39 times higher risk of fracture than controls (*p* = 0.042).

**Table 3:** Logistic regression results for ECA 18 haplotype block 1

**Haplotype**	**Odds ratio**	**t-statistic (Wald test)**	**P-value**	**Frequency in cases**	**Frequency in controls**
GGAGGCTAAA	0.511	15	1 × 10^-4^	0.738	0.856
TAGAACCGGG	1.42	3.66	0.161	0.196	0.130
TGGAATTAAG	3.39	5.41	0.042	0.029	0
GAGAACCGGG	1.8 × 10^11^	2.51	0.286	0.024	0

Background of horse (flat or National Hunt-bred) and sex were fitted as covariates in the haplotype logistic regression option in PLINK, *p*-values were derived using 10,000 permutations.

There is low LD (r^2^ < 0.1) between adjacent haplotype blocks in the ECA 18 region. Haplotype block 1 contains 39.5 kb of the zinc finger protein 804A gene (*ZNF804A*) and the two most significant SNPs in the GWAS are located in this haplotype block, 2.2 kb from the end of the gene. *ZNF804A* has been reported to have a variant associated with schizophrenia in humans [20,21], and regulates expression of genes such as the catechol O-methyl transferase gene (*COMT*) [22] which has been associated with increased fracture risk in males [23]. An elevated risk of fracture has been noted in schizophrenics [24], but no genes directly associated with fracture risk in schizophrenics have previously been reported.

Other candidate genes in ECA 18 haplotype block 3 are integrin alpha-V (*ITGAV*, ECA 18: 63,417,718 – 63,498,794), a receptor binding to a variety of extracellular matrix proteins including osteopontin and bone sialoprotein, the calcitonin receptor-like gene (*CALCRL*, ECA 18: 64,065062 – 64, 102,603), collagen type III alpha 1 (*COL3A1*, ECA 18: 65,487,247 – 65,526,274), collagen type III alpha 2 (*COL3A2*), and collagen type V alpha 2 (*COL5A2*, ECA 18: 65,549,357 – 65,689,370). LD between ECA 18 haplotype blocks 1 and 3 is generally low (r^2^ < 0.3), apart from SNPs BIEC2-417210 and BIEC2-417274 which are in moderate LD (r^2^ = 0.38 – 0.46) with SNPs in block 1. The LD observed may have arisen due to a combination of selected alleles at different genes in this region. For example, there is evidence that racing performance and optimal racing distance in the Thoroughbred horse is influenced by the nearby myostatin (*MSTN*) locus [25-27] and the extent of LD observed in this region may be the result of a selective sweep [28].

### Genetic variance explained by SNPs

**Figure 3:**
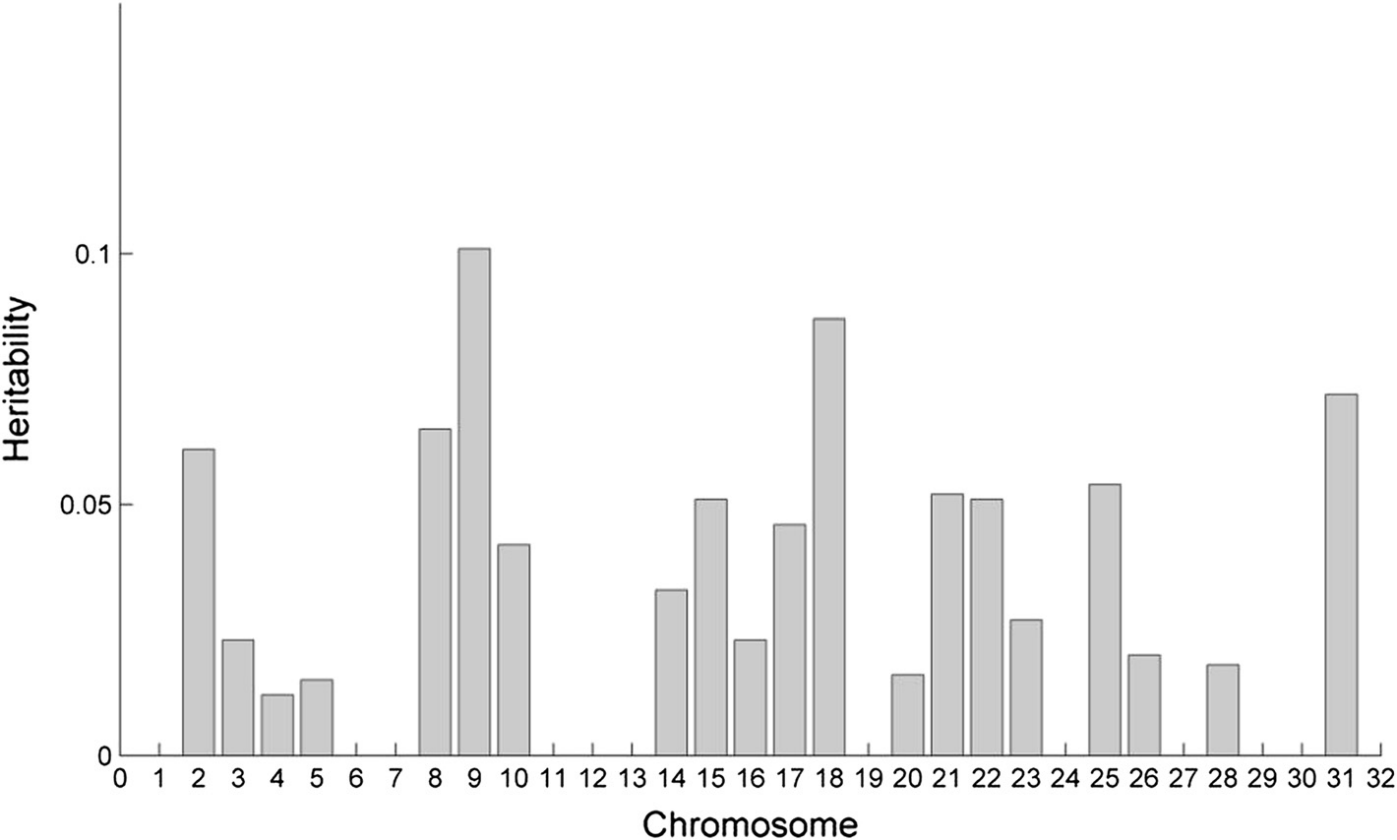
**Heritability of fracture risk by chromosome.** Estimates of the genetic variance explained by SNPs on individual chromosomes were obtained with Restricted Maximum Likelihood (REML) analysis using the GCTA program.

Genetic variance explained by SNPs for fracture risk was estimated to be 0.479 (s.e. 0.124). A log-likelihood of 110.6 for the full model compared with a log-likelihood of 103.4 for the null model (genetic variance σ_g_^2^ = 0) and likelihood ratio test (LRT) of 14.32 (*p* = 0.00015) confirms the variance is significantly different from zero. Genetic variance estimates for each individual chromosome showed significant variance on chromosomes 9, 18, 22 and 31 (Figure 3 and Additional file 5: Table S3). Chromosomes 9 and 18 accounted for the largest genetic variance, around 0.19, followed by chromosomes 22 and 31. Together these chromosomes account for 61.8% of the total estimated genetic variance.

The highest individual chromosome genetic variance estimates correspond with some, but not all, of the chromosomes identified as showing significant association with fracture risk in the genome-wide association study (GWAS). REML analysis to estimate the genetic variances accounts for both genetic relatedness among individuals, through the SNP-based genetic relationship matrix, and population structure [18,19]. In contrast, association methods rely on individuals being unrelated and where there is cryptic relatedness among individuals this can result in an inflation of type I errors or false positives. We have used both approaches in this study, giving increased confidence in chromosomes where the results are concordant. GWAS methods are conservative in their approach, as stringent significance thresholds must be reached before a result is declared significant. Large sample sizes are generally required (> 1000 cases and controls) before these significance thresholds are reached. SNPs that do not reach the significance threshold for a GWAS may, nevertheless, still have genetic effects on the disease. Whether or not they reach the significance threshold depends on the size of the allele effect, the allele frequencies and the degree of linkage disequilibrium between the SNPs and the causal mutations.

## Conclusions

Significant genetic variation for fracture risk in the Thoroughbred horse was detected on chromosomes 9, 18, 22 and 31 using REML analysis. In a related genome-wide association study SNPs on chromosomes 1 and 18 reached genome-wide significance. Several plausible candidate genes involved in bone development are located in these regions. However, the identification of further candidate regions for fracture risk is likely to require larger sample sizes. This study has demonstrated that fracture risk in the horse is heritable and that there is the potential to develop SNP-based estimators for genetic risk of fracture in the Thoroughbred racehorse.

## Competing interests

A patent application has been filed: UK patent application No. 1314131.2.

## Authors’ contributions

TDHP, MV and SCB designed the study. MH, JRN, TDHP and LH collected samples. CS, LYF-C and JES prepared the data and performed quality control analyses. SCB performed statistical analyses. SCB, JES, TDHP, LH, MV contributed to writing the paper. All authors read and approved the final manuscript.

## Supplementary Material

Additional file 1: Table S1 Distribution of fracture locations among the cases. Click here for file

Additional file 2: Table S2 List of additional 78 SNPs genotyped on ECA 18 between 61.89 Mb and 71.17 Mb. Click here for file

Additional file 3: Figure S1 Multidimensional scaling (MDS) plots showing National Hunt and flat-bred horses and fracture cases and controls. Click here for file

Additional file 4: Figure S2 Quantile-quantile (Q-Q) plot obtained after the Cochran-Mantel-Haenszel (CMH) association test. Click here for file

Additional file 5: Table S3 Genetic variance and heritability estimates for fracture risk by chromosome. Click here for file
