# Supplementary material for: A genome-wide association study demonstrates significant genetic variation for fracture risk in Thoroughbred racehorses
Source: BMC Genomics. 2014 Feb 21;15:147. doi: 10.1186/1471-2164-15-147 (PMC4008154; doi:10.1186/1471-2164-15-147)
Supplement: Additional file 1: Table S1 — Distribution of fracture locations among the cases. [file 1471-2164-15-147-S1.doc]

**Table S1**. Distribution of fracture locations among the 269 cases passing genotyping quality control.

| **Bone in which fracture was located** | **No. of cases** |
| --- | --- |
|  |  |
| Lateral condyle of metacarpus/metatarpus III | 103 |
| Medial condyle of metacarpus/metatarpus III | 24 |
| Dorsal cortex of metacarpus/metatarpus III | 24 |
| Lateral and medial condyle of metacarpus/metatarpus III | 5 |
| Proximal phalanx | 62 |
| Proximal sesamoid bones | 27 |
| Carpus | 21 |
| Calcaneus | 3 |
|  |  |
| **Total** | 269 |
