# Supplementary material for: A genome-wide association study demonstrates significant genetic variation for fracture risk in Thoroughbred racehorses
Source: BMC Genomics. 2014 Feb 21;15:147. doi: 10.1186/1471-2164-15-147 (PMC4008154; doi:10.1186/1471-2164-15-147)
Supplement: Additional file 2: Table S2 — List of additional 78 SNPs genotyped on ECA 18 between 61.89 Mb and 71.17 Mb. [file 1471-2164-15-147-S2.doc]

**Table S2**. Additional 78 SNPs genotyped on ECA 18 which were not present on the Equine SNP50 BeadChip.

| **SNP ID** | **ECA** | **Position**  **EquCab2.0** | **Genomic Allele** | **Alternate Allele** | **Discovery Breed(s)** | **Discovery Read(s)** |
| --- | --- | --- | --- | --- | --- | --- |
| BIEC2-438190 | chr18 | 61,891,459 | T | G | Thoroughbred | S261P615RK22.T0 |
| BIEC2-438191 | chr18 | 61,891,676 | G | C | Thoroughbred | S261P615RK22.T0 |
| BIEC2-438193 | chr18 | 61,920,389 | A | C | Thoroughbred | S261P647RI22.T0 |
| BIEC2-438194 | chr18 | 61,920,553 | T | C | Thoroughbred | S261P647RI22.T0 |
| BIEC2-438197 | chr18 | 61,961,892 | A | T | Icelandic | S258P663FK20.T0 |
| BIEC2-438202 | chr18 | 62,007,689 | A | G | Andalusian | S257P629FA4.T0 |
| BIEC2-438205 | chr18 | 62,035,484 | A | G | Icelandic | S258P680FO16.T0 |
| BIEC2-438210 | chr18 | 62,054,710 | T | C | Thoroughbred | S261P6132RP16.T0 |
| BIEC2-438212 | chr18 | 62,115,356 | A | G | Standardbred | S260P629RL10.T0 |
| BIEC2-438214 | chr18 | 62,115,702 | G | A | Standardbred | S260P629RL10.T0 |
| BIEC2-438219 | chr18 | 62,118,403 | C | T | Standardbred | S260P629FL10.T0 |
| BIEC2-438222 | chr18 | 62,118,622 | C | T | Standardbred | S260P629FL10.T0 |
| BIEC2-438227 | chr18 | 62,120,598 | T | C | QuarterHorse | S256P633FA23.T0 |
| BIEC2-438306 | chr18 | 62,794,717 | T | C | Standardbred | S260P628FI11.T0 |
| BIEC2-438408 | chr18 | 63,381,207 | T | C | Andalusian | S257P687FM5.T0 |
| BIEC2-438410 | chr18 | 63,384,365 | T | G | QuarterHorse | S256P6145RM21.T0 |
| BIEC2-438412 | chr18 | 63,384,963 | C | T | Thoroughbred | S261P6106RA12.T0 |
| BIEC2-438416 | chr18 | 63,389,933 | C | G | AkalTeke | S259P695RH9.T0 |
| BIEC2-438418 | chr18 | 63,427,479 | G | C | Andalusian | S257P698FG5.T0 |
| BIEC2-438419 | chr18 | 63,430,922 | T | A | Andalusian | S257P698RG5.T0 |
| BIEC2-438420 | chr18 | 63,454,811 | A | T | Standardbred | S260P693RJ19.T0 |
| BIEC2-438423 | chr18 | 63,465,870 | G | C | Andalusian | S257P627RK6.T0 |
| BIEC2-438425 | chr18 | 63,485,771 | C | T | Andalusian | S257P6120RB1.T0 |
| BIEC2-438427 | chr18 | 63,486,030 | G | C | Andalusian | S257P6120RB1.T0 |
| BIEC2-438431 | chr18 | 63,536,020 | T | C | Genomic_Twilight | Twilight |
| BIEC2-438432 | chr18 | 63,536,194 | A | G | Genomic_Twilight | Twilight |
| BIEC2-438433 | chr18 | 63,540,466 | G | A | AkalTeke | S259P611FH17.T0 |
| BIEC2-438435 | chr18 | 63,549,121 | G | T | Arabian | S255P634RC23.T0 |
| BIEC2-438520 | chr18 | 64,043,949 | A | G | Standardbred | S260P638FI18.T0 |
| BIEC2-438522 | chr18 | 64,044,397 | A | G | Standardbred | S260P638FI18.T0 |
| BIEC2-438523 | chr18 | 64,046,952 | C | G | Standardbred | S260P638RI18.T0 |
| BIEC2-438524 | chr18 | 64,047,063 | C | T | Standardbred | S260P638RI18.T0 |
| BIEC2-438526 | chr18 | 64,118,925 | C | T | AkalTeke | S259P6119FB4.T0 |
| BIEC2-438527 | chr18 | 64,122,914 | C | T | AkalTeke | S259P6119RB4.T0 |
| BIEC2-438534 | chr18 | 64,233,475 | T | C | Andalusian | S257P635FP16.T0 |
| BIEC2-438535 | chr18 | 64,233,776 | G | C | Andalusian | S257P635FP16.T0 |
| BIEC2-438536 | chr18 | 64,240,243 | C | A | Arabian | S255P685FP21.T0 |
| BIEC2-438541 | chr18 | 64,252,426 | T | C | Thoroughbred | S261P68FD17.T0 |
| BIEC2-438545 | chr18 | 64,366,081 | T | G | Standardbred | S260P6107RD6.T0 |
| BIEC2-438694 | chr18 | 65,194,006 | C | T | AkalTeke | S259P688FM18.T0 |
| BIEC2-438696 | chr18 | 65,248,091 | G | A | Arabian | S255P680RM11.T0 |
| BIEC2-438697 | chr18 | 65,248,224 | A | G | Arabian | S255P680RM11.T0 |
| BIEC2-438720 | chr18 | 65,443,592 | T | A | Thoroughbred | S261P618RA3.T0 |
| BIEC2-438722 | chr18 | 65,454,331 | G | A | QuarterHorse | S256P655FA19.T0 |
| BIEC2-438725 | chr18 | 65,514,599 | C | T | Thoroughbred | S261P6121FN18.T0 |
| BIEC2-438726 | chr18 | 65,520,546 | A | G | Icelandic | S258P6128RH14.T0 |
| BIEC2-438728 | chr18 | 65,539,461 | C | T | QuarterHorse | S256P667RL17.T0 |
| BIEC2-438731 | chr18 | 65,557,759 | C | T | Standardbred | S260P6100FA24.T0 |
| BIEC2-438732 | chr18 | 65,560,562 | C | T | Arabian | S255P64RB9.T0 |
| BIEC2-438733 | chr18 | 65,564,741 | G | A | Arabian | S255P64FB9.T0 |
| BIEC2-438908 | chr18 | 66,393,104 | C | T | Arabian | S255P6116FD2.T0 |
| BIEC2-438909 | chr18 | 66,393,299 | A | C | Arabian | S255P6116FD2.T0 |
| BIEC2-438910 | chr18 | 66,393,815 | T | A | Icelandic | S258P656FC11.T0 |
| BIEC2-438912 | chr18 | 66,428,044 | G | A | Standardbred | S260P676RD1.T0 |
| BIEC2-438913 | chr18 | 66,428,610 | G | A | Standardbred | S260P676RD1.T0 |
| BIEC2-438914 | chr18 | 66,476,302 | T | C | Thoroughbred | S261P696FN8.T0 |
| BIEC2-438925 | chr18 | 66,511,568 | T | G | Icelandic | S258P65FI18.T0 |
| BIEC2-438927 | chr18 | 66,513,576 | G | A | Icelandic | S258P667FL7.T0 |
| BIEC2-438928 | chr18 | 66,515,465 | C | T | Icelandic | S258P65RI18.T0 |
| BIEC2-438929 | chr18 | 66,517,329 | C | T | Icelandic | S258P667RL7.T0 |
| BIEC2-438931 | chr18 | 66,529,056 | T | A | Thoroughbred | S261P66RD24.T0 |
| BIEC2-438932 | chr18 | 66,539,874 | C | T | Arabian | S255P6115FO19.T0 |
| BIEC2-438934 | chr18 | 66,540,780 | A | G | Andalusian | S257P685FK9.T0 |
| BIEC2-438969 | chr18 | 66,732,110 | A | G | Thoroughbred | S261P6100FP1.T0 |
| BIEC2-438973 | chr18 | 66,755,421 | T | C | AkalTeke | S259P672FA18.T0 |
| BIEC2-439067 | chr18 | 67,257,891 | G | A | Arabian | S255P656FK8.T0 |
| BIEC2-439070 | chr18 | 67,261,123 | A | C | Arabian,Icelandic | S255P656RK8.T0,  S258P664RH1.T0 |
| BIEC2-439071 | chr18 | 67,265,071 | A | T | Icelandic | S258P664FH1.T0 |
| BIEC2-439073 | chr18 | 67,320,937 | G | A | Standardbred | S260P668FG20.T0 |
| BIEC2-439074 | chr18 | 67,359,676 | G | T | Thoroughbred | S261P63FB10.T0 |
| BIEC2-439076 | chr18 | 67,377,787 | A | C | Genomic_Twilight | Twilight |
| BIEC2-439080 | chr18 | 67,438,643 | T | C | Andalusian | S257P667FG9.T0 |
| BIEC2-439081 | chr18 | 67,444,793 | C | T | Arabian | S255P636RA18.T0 |
| BIEC2-439082 | chr18 | 67,449,955 | G | A | Arabian | S255P695FA15.T0 |
| BIEC2-439083 | chr18 | 67,451,900 | G | A | Andalusian | S257P664RK7.T0 |
| BIEC2-439084 | chr18 | 67,457,641 | C | T | Icelandic | S258P672FH18.T0 |
| BIEC2-418029 | chr18 | 71,132,863 | G | T | AkalTeke | S259P628RN8.T0 |
| BIEC2-418035 | chr18 | 71,164,790 | T | C | Arabian | S255P649RK22.T0 |
|  |  |  |  |  |  |  |
