# Supplementary material for: A genome-wide association study demonstrates significant genetic variation for fracture risk in Thoroughbred racehorses
Source: BMC Genomics. 2014 Feb 21;15:147. doi: 10.1186/1471-2164-15-147 (PMC4008154; doi:10.1186/1471-2164-15-147)
Supplement: Additional file 3: Figure S1 — Multidimensional scaling (MDS) plots showing National Hunt and flat-bred horses and fracture cases and controls. [file 1471-2164-15-147-S3.doc]

**Figure S1**. a) Multidimensional scaling plot showing National Hunt and flat-bred horses.


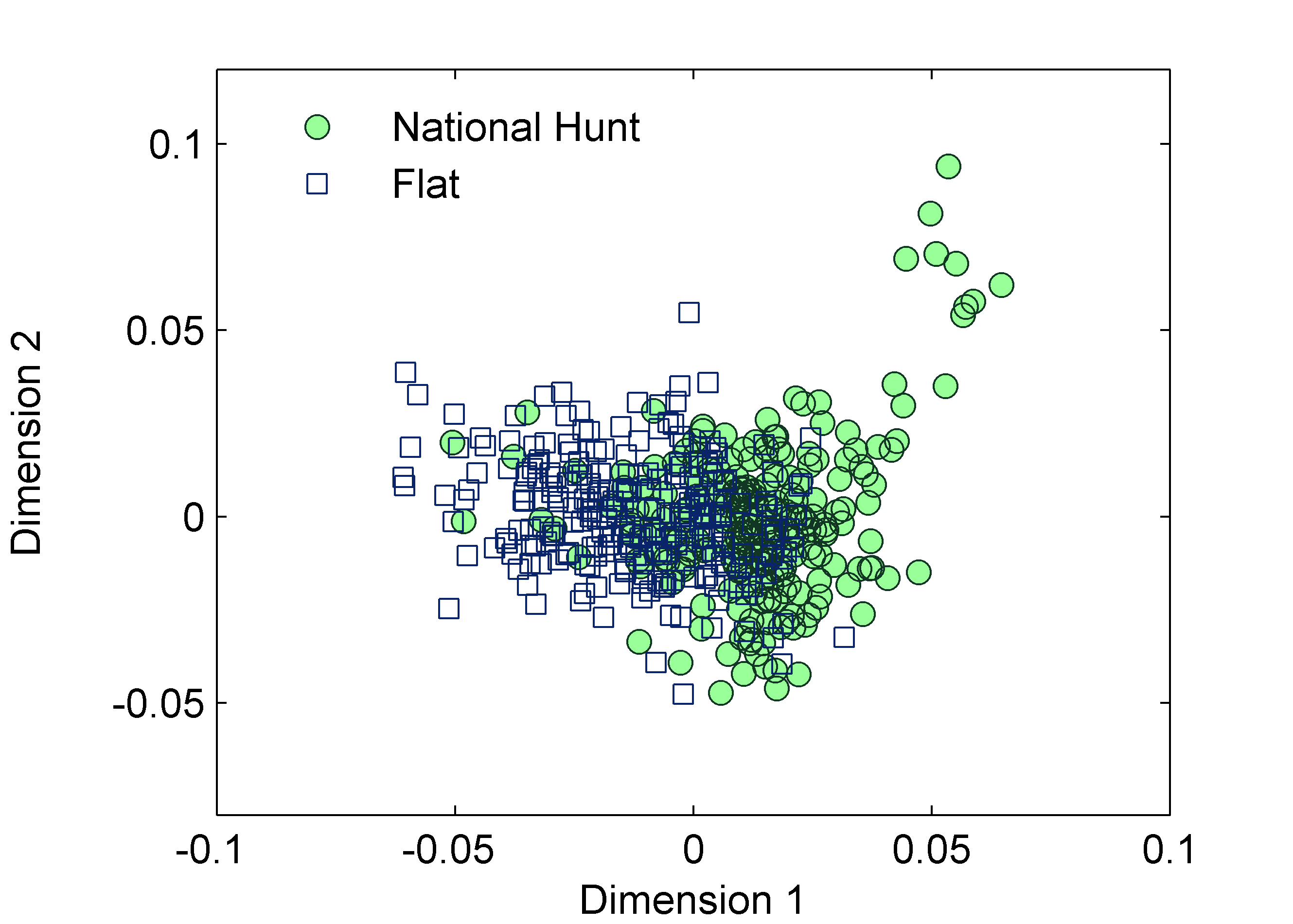


b) Multidimensional scaling plot showing fracture cases and controls.

**
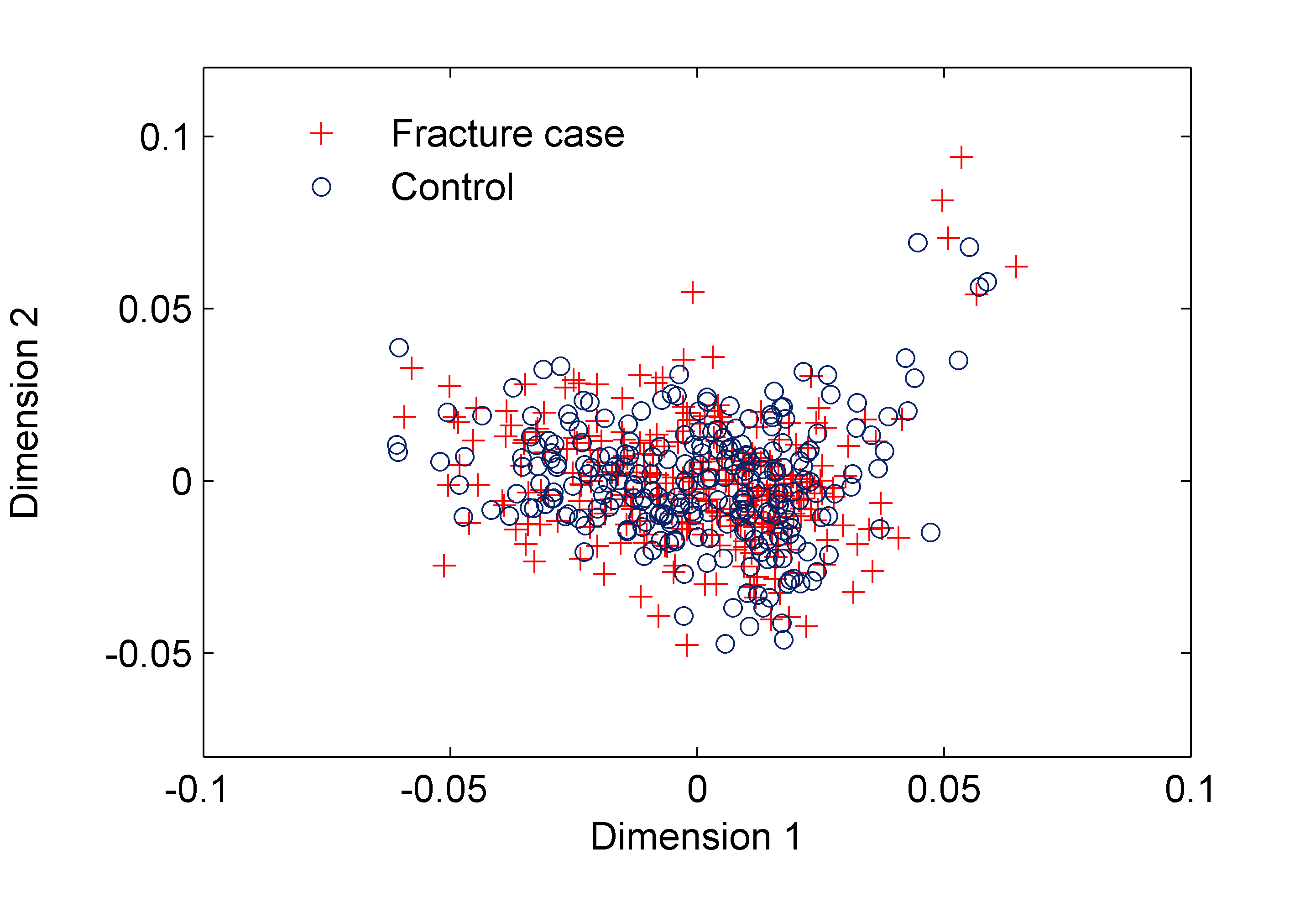
**

Multi-dimensional scaling, based on an identity-by-state (IBS) distance matrix, was used to visualise clustering among individuals. A permutation test for between group IBS differences showed a significant (*p* < 2 x 10^-5^) degree of similarity (IBS) between cases and controls, although IBS within case and control groups was not significant. There was also a significant degree of similarity between flat-bred and National Hunt-bred horses (*p* < 1 x 10^-5^), with evidence for more similarity (clustering) within flat-bred and National Hunt-bred groups than between the groups (within-group *p* < 1 x 10^-5^, between-group *p* < 7 x 10^-5^).

c) Multidimensional scaling plot showing relationships (coancestry) based on pedigree among National Hunt (NH) and flat sires in the UK in 2012


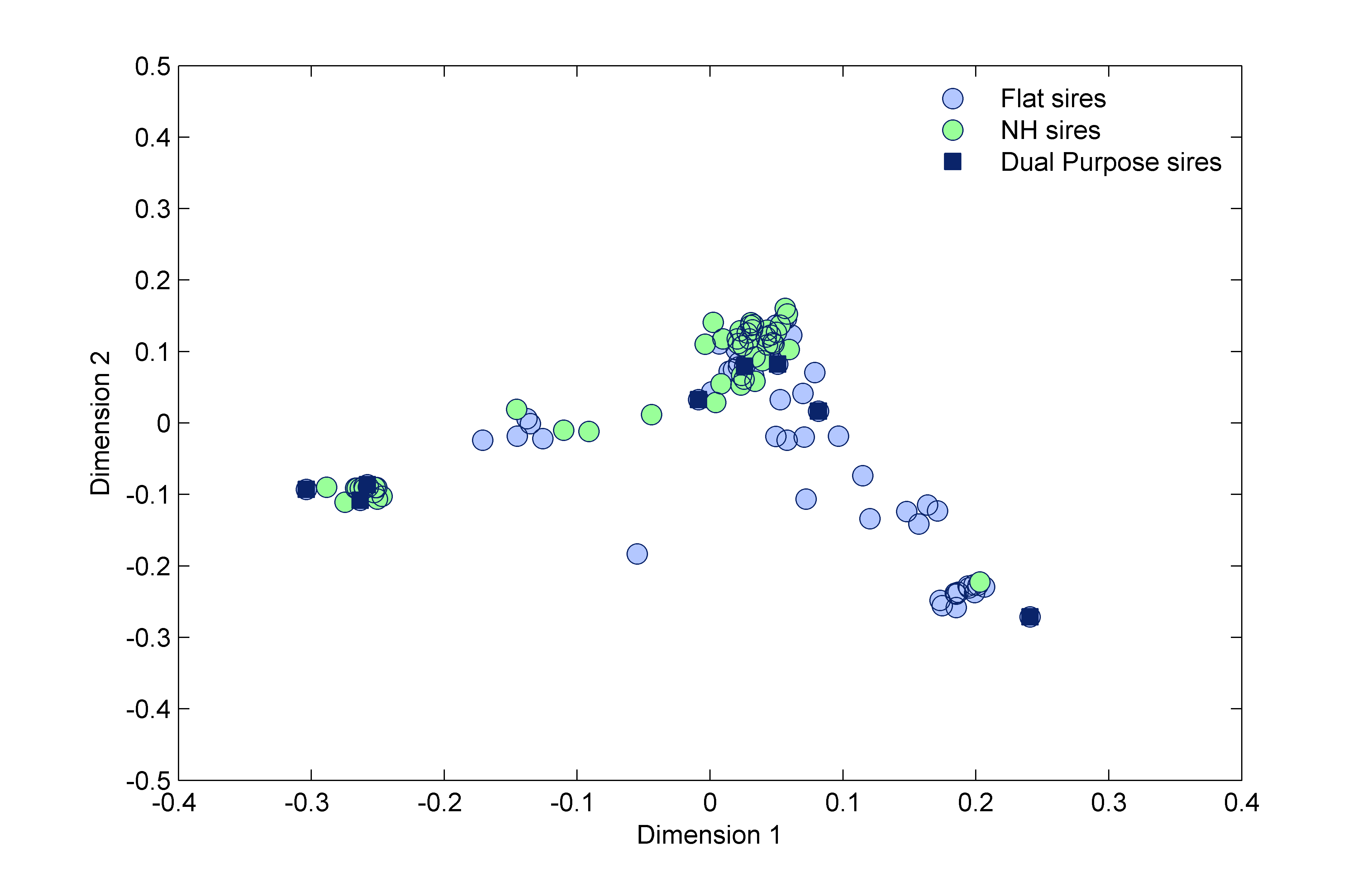


Coancestry was calculated between the top 60 NH sires (ranked by offspring earnings in 2012) and top 60 flat sires using at least 7 generations of pedigree. Eight sires appeared on both lists and are designated dual-purpose sires on the plot. The mean coancestry among NH and flat sires was 0.0295, minimum coancestry was 0.0024 and maximum coancestry 0.276. The existence of non-zero coancestry between flat and NH sires confirms there is gene flow between the two populations. The multidimensional scaling plot illustrates the family clustering within NH or flat type but also suggests there is some differentiation between family lines as to whether they are used to produce flat or NH horses.
