# Supplementary material for: A genome-wide association study demonstrates significant genetic variation for fracture risk in Thoroughbred racehorses
Source: BMC Genomics. 2014 Feb 21;15:147. doi: 10.1186/1471-2164-15-147 (PMC4008154; doi:10.1186/1471-2164-15-147)

**Figure S2**. The quantile-quantile (Q-Q) plot obtained after the Cochran-Mantel-Haenszel (CMH) association test.


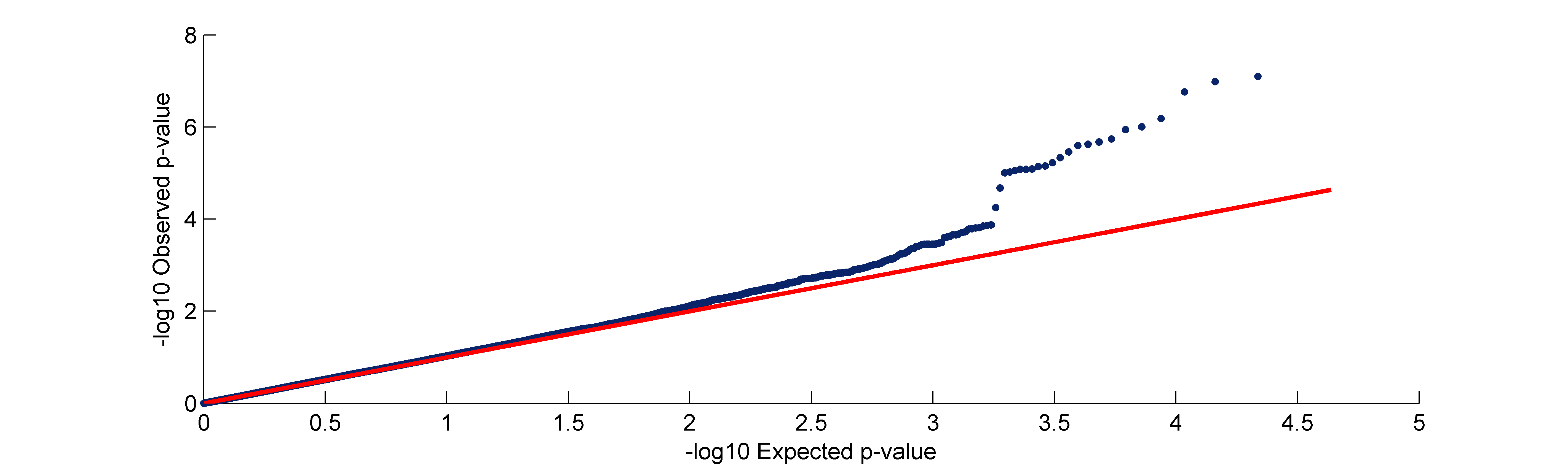

Supplement: Additional file 4: Figure S2 — Quantile-quantile (Q-Q) plot obtained after the Cochran-Mantel-Haenszel (CMH) association test. [file 1471-2164-15-147-S4.doc]
