# Supplementary material for: A genome-wide association study demonstrates significant genetic variation for fracture risk in Thoroughbred racehorses
Source: BMC Genomics. 2014 Feb 21;15:147. doi: 10.1186/1471-2164-15-147 (PMC4008154; doi:10.1186/1471-2164-15-147)
Supplement: Additional file 5: Table S3 — Genetic variance and heritability estimates for fracture risk by chromosome. [file 1471-2164-15-147-S5.doc]

**Table S3**. Genetic variance and heritability estimates for fracture risk by chromosome. Model fitted includes as co-variates sex and first 20 eigenvectors (to account for population stratification). Estimate of Vp is 0.236 (s.e. 0.015).

| **Chromosome** | **Va** | **s.e.** | **h^2^** | **s.e.** | **LogL** | **LRT** |
| --- | --- | --- | --- | --- | --- | --- |
| 1 | 0.000 | 0.009 | 0.000 | 0.037 | 103.39 |  |
| 2 | 0.015 | 0.011 | 0.061 | 0.046 | 104.45 |  |
| 3 | 0.005 | 0.009 | 0.023 | 0.039 | 103.56 |  |
| 4 | 0.003 | 0.009 | 0.012 | 0.037 | 103.43 |  |
| 5 | 0.004 | 0.008 | 0.015 | 0.034 | 103.51 |  |
| 6 | 0.000 | 0.006 | 0.000 | 0.027 | 103.39 |  |
| 7 | 0.000 | 0.007 | 0.000 | 0.029 | 103.39 |  |
| 8 | 0.016 | 0.012 | 0.065 | 0.049 | 104.23 |  |
| 9 | 0.025 | 0.013 | 0.101 | 0.050 | 106.91 | 7.04 (*p* = 0.008) |
| 10 | 0.010 | 0.009 | 0.042 | 0.039 | 104.14 |  |
| 11 | 0.000 | 0.006 | 0.000 | 0.027 | 103.39 |  |
| 12 | 0.000 | 0.006 | 0.000 | 0.026 | 103.39 |  |
| 13 | 0.000 | 0.006 | 0.000 | 0.025 | 103.39 |  |
| 14 | 0.008 | 0.008 | 0.033 | 0.015 | 104.39 |  |
| 15 | 0.012 | 0.010 | 0.051 | 0.042 | 104.35 |  |
| 16 | 0.005 | 0.008 | 0.023 | 0.036 | 103.61 |  |
| 17 | 0.012 | 0.009 | 0.046 | 0.039 | 104.33 |  |
| 18 | 0.021 | 0.011 | 0.087 | 0.044 | 107.27 | 7.76 (*p* = 0.005) |
| 19 | 0.000 | 0.006 | 0.000 | 0.025 | 103.39 |  |
| 20 | 0.004 | 0.007 | 0.016 | 0.028 | 103.59 |  |
| 21 | 0.012 | 0.009 | 0.052 | 0.036 | 104.97 | 3.16 (*p* = 0.075) |
| 22 | 0.012 | 0.008 | 0.051 | 0.034 | 105.35 | 3.92 (*p* = 0.048) |
| 23 | 0.006 | 0.008 | 0.027 | 0.032 | 103.85 |  |
| 24 | 0.000 | 0.006 | 0.000 | 0.028 | 103.36 |  |
| 25 | 0.013 | 0.009 | 0.054 | 0.037 | 105.19 | 3.60 (*p* = 0.058) |
| 26 | 0.005 | 0.006 | 0.020 | 0.026 | 103.87 |  |
| 27 | 0.000 | 0.006 | 0.000 | 0.028 | 103.39 |  |
| 28 | 0.004 | 0.007 | 0.018 | 0.028 | 103.65 |  |
| 29 | 0.000 | 0.005 | 0.000 | 0.022 | 103.39 |  |
| 30 | 0.000 | 0.005 | 0.000 | 0.019 | 103.39 |  |
| 31 | 0.017 | 0.009 | 0.072 | 0.038 | 106.93 | 7.08 (*p* = 0.008) |
| X | 0.000 | 0.007 | 0.000 | 0.031 | 103.39 |  |
